# Community paramedic point of care testing: validity and usability of two commercially available devices

**DOI:** 10.1186/s12873-019-0243-4

**Published:** 2019-05-02

**Authors:** Ian E. Blanchard, Ryan Kozicky, Dana Dalgarno, Justin Simms, Stacy Goulder, Tyler S. Williamson, Susan Biesbroek, Lenore Page, Karen Leaman, Suzanne Snozyk, Lyle Redman, Keith Spackman, Christopher J. Doig, Eddy S. Lang, Gerald Lazarenko

**Affiliations:** 1 Alberta Health Services, Emergency Medical Services, 10101 Southport Road SW, Calgary, AB T2W 3N1 Canada; 2 Alberta Health Services, Human Factors, Calgary, Alberta Canada; 3 University of Calgary, Cumming School of Medicine, Calgary, Alberta Canada; 4 Calgary Laboratory Services, Calgary, Alberta Canada

**Keywords:** Point-of-care systems, Emergency medical services, Paramedic, Electrolytes, Creatinine, Hemoglobin

## Abstract

**Background:**

Community Paramedics (CPs) require access to timely blood analysis in the field to guide treatment and transport decisions. Point of care testing (POCT), as opposed to laboratory analysis, may offer a solution, but limited research exists on CP POCT. The purpose of this study was to compare the validity of two devices (Abbott i-STAT® and Alere epoc®) by CPs in the community.

**Methods:**

In a CP programme responding to 6000 annual patient care events, a split sample validation of POCT against traditional laboratory analysis for seven analytes (sodium, potassium, chloride, creatinine, hemoglobin, hematocrit, and glucose) was conducted on a consecutive sample of patients. The difference of proportion of discrepant results between POCT and laboratory was compared using a two sample proportion test. Usability was analysed by survey of CP experience, a linear mixed effects model of Systems Usability Scale (SUS) adjusted for CP clinical and POCT experience, an expert heuristic evaluation of devices, a review of device-logged errors, and coded observations of POCT use during quality control testing.

**Results:**

Of 1649 episodes of care screened for enrollment, 174 required a blood draw, with 108 episodes (62.1%) enrolled from 73 participants. Participants had a mean age of 58.7 years (SD16.3); 49% were female. In 4 of 646 (0.6%) comparisons, POCT reported a critical value but the laboratory did not; with no statistically significant (*p* = 0.323) difference between i-STAT® (0.9%;95%CI:0.0,1.9%) compared with epoc® (0.3%;95%CI:0.0,0.9%). There were no instances of the laboratory reporting a critical value when POCT did not. In 88 of 1046 (8.4%) comparisons the a priori defined acceptable difference between POCT and the laboratory was exceeded; occurring more often in epoc® (10.7%;95%CI:8.1,13.3%) compared with i-STAT® (6.1%;95%CI:4.1,8.2%)(*p* = 0.007). Eighteen of 19 CP surveys were returned, with 11/18 (61.1%) preferring i-STAT® over epoc®. The i-STAT® had a higher mean SUS score (higher usability) compared with epoc® (84.0/100 vs. 59.6/100; *p* = 0.011). There were no statistically significant differences in device logged errors between i-STAT® and epoc® (*p* = 0.063).

**Conclusions:**

CP programmes can expect clinically valid results from POCT. Device usability assessments should be considered with any local implementation as the two POCT systems have different strengths.

**Electronic supplementary material:**

The online version of this article (10.1186/s12873-019-0243-4) contains supplementary material, which is available to authorized users.

## Background

The traditional role of Emergency Medical Services (EMS) systems is to respond to emergency calls. Paramedics’ traditional role in EMS is changing, including where Community Paramedics (CPs) provide a bridge between the hospital and the community by offering specialized primary care services for individuals with chronic diseases or difficulty accessing traditional healthcare services. While there is heterogeneity in the structure and process of CP programmes, in general these programmes focus on high needs patients such as the frail elderly. [1] CPs receive training in addition to their formative training, and generally have a broader scope of practice compared with regular duty paramedics. CP care may prevent patient transport to an acute care facility, which may have positive implications for the patient’s physical and mental health as patients can stay at home, and positive implications for the health system especially overcrowding of Emergency Departments. [2, 3]

One of the challenges of providing care to these patients in the community is timely access to diagnostic tests such as blood analyses. Presently the primary option for many CP programmes is to collect blood specimens and transport them to a laboratory service for analysis. The process involves the CP collecting a blood sample in the community, transporting the sample to a blood testing laboratory, and following-up on results, often hours later. This process is resource intensive, presents multiple opportunities for misidentification of patients or results, and may delay timely treatment. An alternative process for CP programmes may be point of care testing (POCT).

POCT technology has advanced considerably in the last decade, resulting in the commercial availability (at the time of this study design) of two portable devices that can provide a variety of blood tests quickly at the patient’s bed side from a venous blood sample (Abbott i-STAT® and Alere epoc®).

A systematic review completed in 2013 on CP care did not identify any peer reviewed studies that assessed the use of POCT technology in this setting, although technology assessment was not the explicit purpose of the review. [1] A number of studies, however, have reported the use of POCT in EMS responses by non-CP ground crews. [4–6] One of the studies did not explicitly compare the results to laboratory values, and one study assessed the i-STAT® troponin I. [4, 5] The final study assessed sodium, potassium, chloride, blood urea nitrogen, glucose, hematocrit and hemoglobin from i-STAT® split sample tests performed in a moving ambulance, to those on the same device in the Emergency Department. [6] This study found correlation (r-values) of greater than 0.89 for all tests. We are unaware of any published peer-reviewed studies that assessed the epoc® device in the EMS setting, described either device in the CP setting, or contrasted the usability of either device. One study done in cardiopulmonary bypass inpatients found the average correlation for nine different hematologic tests to be r = 0.97 +/− 0.023 when epoc® was compared with laboratory analysis, and r = 0.97 +/− 0.029 when epoc® was compared with the i-STAT®. [7] This suggests that these two devices are functionally similar in the hands of laboratory personnel in a controlled environment.

The purpose of this study was to assess the validity of two commercially available devices (Abbott i-STAT® and Alere epoc®) in the CP setting against the reference standard of laboratory analysis and compare the usability of these devices by CPs.

## Methods

### Study setting

This study was conducted in a mature CP programme that responds to approximately 6000 patient care events per year. Patients can be generally described as medically fragile and seen in a home setting (e.g., continuing care facility, private residence, and homeless shelter). At the time of this study there were 19 active CPs in the programme using five vehicles that cannot convey patients and one that can. CPs must be registered as an Advanced Care Paramedic with the Alberta College of Paramedics, and have at least five years of clinical experience. [8] In addition to their formative paramedic training, CPs receive 21 days of additional training on assessment and treatment skills specific to this patient population. These skills consist of geriatric medicine, social determinants of health, advanced cardiopulmonary assessments, additional pharmacology (in particular antibiotics), urinary catheterization, wound closure (sutures, staples, adhesives), accessing central venous access devices, specimen collection, which includes wound, throat, and nasopharyngeal swabs, and blood and urine specimens.

In routine practice CPs draw blood specimens and transport the sample to twelve different laboratory service locations for analysis. The CP will then follow-up on results several hours later, discuss results with a physician, and if required re-visit the patient to implement or modify a treatment plan.

### Study design and experimental protocol for device validation

Consecutive patients meeting inclusion criteria were enrolled by CPs into a modified single subject (split-sample) study between September 1 and November 30, 2016. Inclusion criteria consisted of patients who had sufficient capacity to be their own decision maker, age was greater than or equal to 18 years, at least one study analyte was ordered for testing, and the patient was able to provide informed consent. Patients were not excluded if they already had been consented into the study. In other words, one patient may have been enrolled multiple times in the study if they had multiple episodes of care that required a blood draw.

After informed consent, a blood draw was carried out and the specimen transported for laboratory blood testing in a “BD vacutainer PST tube” with 56 units of lithium heparin as per routine practice. On scene a portion of the drawn blood was also used for POCT testing (split-sample).

POCT testing involved the use of both i-STAT® and epoc® devices. The analytes sodium (Na), potassium (K), chloride (Cl), creatinine (Crea), hemoglobin (Hgb), hematocrit (Hct), and glucose (Glu) were included in the study. The rationale for choosing these particular analytes was the high frequency of occurrence in the CP programme and availability on each of the test cartridges or cards for the two POCT devices.

### Study design and experimental protocol for device usability

For comparing device usability, four assessment methods were used to increase the validity of the collected data. They were an online preference and feedback survey for CPs (with a standardized device usability survey embedded), usability testing, device-logged error analysis and heuristic evaluation of the two devices. The latter three methods elucidate upon and can objectively validate the online survey responses.

The online survey was developed to gather CP experiences, preference, and feedback regarding both POCT devices. The survey was pilot tested on a CP team lead and one of the investigators and refined accordingly prior to sending to all CPs involved in the study (December 2 to 31, 2016).

To reduce the effect of device order influencing survey responses, participants were randomly assigned the survey order for each device (either i-STAT® or epoc® first) using R sample command. [9, 10] Answer choices to the device preference questions were presented in random order using the Survey software platform answer randomization command. [11]

A portion of the survey required participants to complete the Systems Usability Scale (SUS) for each device. The SUS is a validated reliable measuring scale of technology learnability and usability. The scores are normalized and can be compared to a benchmark of quartile ranges, acceptability ranges and adjective ratings. [12]

In addition, two Human Factors consultants reviewed device usability with both heuristic evaluation and usability testing methodologies. Heuristic evaluation is a method of device interface evaluation that uses broad categories of design principles to systematically evaluate usability problems. [13] The consultants worked through a number of tasks on the two devices, to identify and evaluate unique design issues and features associated with each of the device’s respective interfaces.

Usability testing was completed by analysing video from the CP’s performing quality control (QC) procedures outlined below. Three observation sessions were used to video record six CPs using the devices. The observations occurred at weeks nine and 10 of exposure to the devices. Participants were video recorded by researchers standing in the room where QC testing normally occurred. Any device errors, including test card or cartridge errors that were encountered, issues running the tests, steps missed and feedback from the staff were incorporated into the human factors review.

### Ethical considerations

The study was approved by the University of Calgary, Conjoint Health Research Ethics Board (REB16–1000). Two populations were identified as participants in this study, the patient and the CP. Each population provided written informed consent as a condition of enrollment into the study.

### Study and device training

CPs received one day (eight hours) of training in the week prior to the start of the study. The curriculum included training on the operation of i-STAT® and epoc® devices and troubleshooting strategies. CPs also received an overview of the research study, ethics, consent procedures, additional equipment, documentation and data collection. Since drawing blood was already in the CP scope of practice and routinely being performed, no additional training was necessary. Each CP received an additional two-hour, device quality control testing training session. While an optimal process for using two POCT devices during a patient event was suggested to CP participants, it was left to each individual CP on how they managed both devices as long as both devices were used as closely as possible to each other.

### Device preparation and maintenance

Six i-STAT® and six epoc® devices were purchased and systematically tested prior to use in the study. The devices, associated test cards or cartridges, and analytes underwent initial laboratory validation using split sample testing of patient blood comparatives to the laboratory reference instruments, with in run and day to day precision testing using liquid QC solutions and calculation verification tests using liquid calculation verification solutions as per standards set by the laboratory service that works with the CP programme. All devices passed the validation, quality control, and calculation verification testing.

While in service, all devices were housed in a temperature controlled and shock resistant environment. Test cartridges for i-STAT® and test cards for epoc® were also stored in the temperature controlled containers. Temperature monitors were placed on the inside and outside of the device containers to ensure an operating temperature of between 18 °C and 30 °C. All QC, calculation verification solutions and additional i-STAT® test cartridges were stored in two fridges that were both temperature monitored throughout the study period. Additional epoc® test cards were stored at room temperature. Devices underwent weekly QC testing and if applicable daily electronic simulation testing as per the manufacturers’ and local laboratory recommendations.

### Analysis

For the device validation objective, a sample of at least 100 patients was the target to provide a margin of error for point estimates of 6.2% on a 95% confidence interval assuming at least a 10% prevalence for out-of-range blood results and a targeted sensitivity of 99% for the device.

Data were downloaded from the two POCT devices and linked to the appropriate electronic patient care records (ePCR) and laboratory values. Data in the ePCR were verified for missing data shortly after the patient contact, and if applicable sent to the author of the ePCR for final completion. All data were manually entered into a Microsoft Excel spreadsheet by one investigator and independently verified by a research associate. Each patient and CP was given a unique study identifier as was each event. All identifying patient data were then removed and the data analyzed using Stata version 11 (Statacorp, College Station, Texas). Descriptive data are reported as means and standard deviations for normally distributed data, or medians and inter-quartile ranges for data that clearly diverge from normality.

POCT results were compared with the reference standard laboratory values using the methods described by Bland and Altman (2009). [14] Critical range values, defined as values for which the analyte result was considered clinically abnormal, and acceptable comparative ranges, defined as the accepted deviation that a POCT can have from the reference standard of laboratory analysis, were based on local laboratory standards and specified a priori (Table 1).

**Table 1:** Summary of critical range values and acceptable comparative ranges by analyte

Analyte	Critical Range	Laboratory to POCT Acceptable Comparative Range
Sodium (Na)	< 120 and > 155 mmol/L	−4 to 4 mmol/L
Potassium (K)	< 2.5 and > 6 mmol/L	−0.3 to 0.3 mmol/L
Chloride (Cl)	n/a	−5 to 5%
Creatinine (Crea)	n/a	−30 to 30 umol/L
Hematocrit (Hct)	n/a	−6 to 6%
Hemoglobin (Hgb)	< 70 g/L	n/a
Glucose (glu)	< 2.6 and > 24.9 mmol/L	< 5 mmol/L: −0.3 to 0.3 mmol/L ≥ 5 mmol/L: −10 to 10%

Note: *POCT* Point of care testing device

The proportion of out-of-range results between i-STAT® and laboratory and epoc® and laboratory were compared by a two sample test of proportion. A Chi-squared test and logistic regression with a Wald test were used to explore if one device contributed more out-of-range results compared with others.

For the contrasting device usability objective, the SUS analysis consisted of a linear regression mixed effects model. The participants were considered as a random intercept effect taking into account their paramedic experience, experience in this specific CP programme and previous exposure to the devices in a work environment.

All statistical tests were considered significant at the 0.05 level.

## Results

### Device validation

**Fig. 1:**
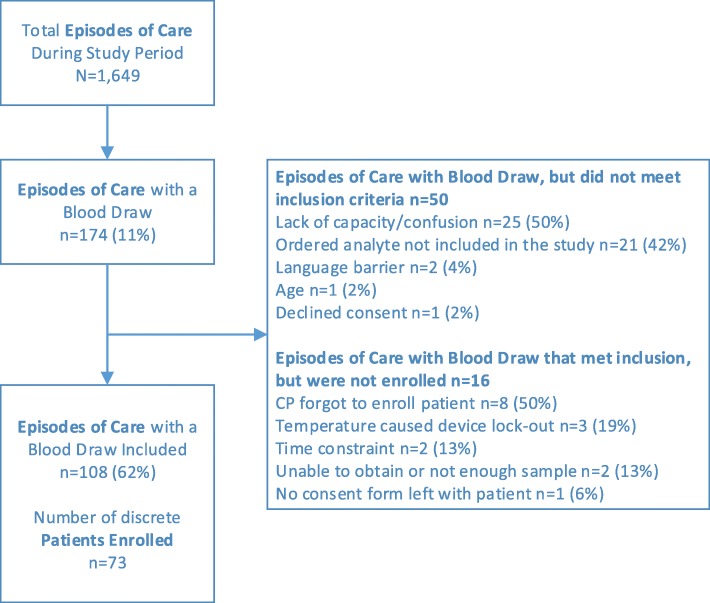
Study enrollment

Of 1649 episodes of care screened for enrollment, 174 episodes of care had a blood draw, with 108 episodes of care enrolled in the study, from 73 participants (Fig. 1). Participants had a mean age of 58.7 years (SD 16.3), and 49% were female. The mean time to transport a specimen to the laboratory was 19.7 min (SD 14.1; 95% CI 17.0, 22.4). The mean time between POCT device result, and the result from the reference laboratory was 129.7 min (SD 169.7; 95% CI 96.9, 162.6).

In 4 of 646 (0.6%) individual comparisons between i-STAT® and laboratory and epoc® and laboratory, POCT reported a critical value but the laboratory did not; occurring more often in i-STAT® (0.9%; 95%CI: 0.0, 1.9%) compared with epoc® (0.3%; 95%CI: 0.0, 0.9%), although these results were not statistically significant (*p* = 0.323) (Table 2). There were no instances of the laboratory reporting a critical value when POCT did not. The discrepant results occurred entirely in the Na and K analytes, with no discrepant results reported for other analytes.

**Table 2:** Summary of disagreements in critical range and values outside of acceptable comparative range between laboratory and POCT by analyte and manufacturer

Critical Range Disagreement				
Analyte	Lab to i-STAT	Lab to epoc	epoc to i-STAT	Total
Sodium	1/101	1/102	2/101	4/304 (1.3%)
Potassium	2/101	0/101	1/100	3/302 (1.0%)
Chloride	n/a	n/a	n/a	n/a
Creatinine	n/a	n/a	n/a	n/a
Hematocrit	n/a	n/a	n/a	n/a
Hemoglobin	0/83	0/84	0/100	0/267 (0.0%)
Glucose				
< 5 mmol/L	0/8	0/8	0/8	0/24 (0.0%)
≥ 5 mmol/L	0/29	0/29	0/92	0/150 (0.0%)
Total	3/322 (0.9%)	1/324 (0.3%)	3/401 (0.7%)	7/1047 (0.7%)
	p = 0.323*			
Outside of the Laboratory to POCT Acceptable Comparative Range				
Analyte	Lab to i-STAT	Lab to epoc	epoc to i-STAT	Total
Sodium	2/101	2/102	1/101	5/304 (1.6%)
Potassium	10/101	9/101	1/100	20/302 (6.6%)
Chloride	5/101	14/101	10/100	29/302 (9.6%)
Creatinine	4/100	10/98	7/98	21/296 (7.1%)
Hematocrit	0/83	17/84	3/100	20/267 (7.5%)
Hemoglobin	n/a	n/a	n/a	n/a
Glucose				
< 5 mmol/L	5/8	2/8	3/8	10/24 (41.7%)
≥ 5 mmol/L	6/29	2/29	13/92	21/150 (14.0%)
Total	32/523 (6.1%)	56/523 (10.7%)	38/599 (6.3%)	126/1645 (7.7%)
	*p* = 0.007*			

Note: *POCT* Point of care testing device

*Two sample test of proportions between i-STAT® compared with laboratory, and epoc® compared with laboratory

In 88 of 1046 (8.4%) individual comparisons between i-STAT® and laboratory and epoc® and laboratory, the a priori defined acceptable difference between POCT and the laboratory was exceeded; occurring more often in epoc® (10.7%; 95%CI: 8.1,13.3%) compared with i-STAT® (6.1%; 95%CI: 4.1,8.2%)(*p* = 0.007)(Table 2). When the i-STAT® to laboratory is compared with epoc® to laboratory, there are similar levels of agreement for Na and K. However, the epoc® has almost three times the number of out-of-range results for Cl, and twice the number for Crea compared with i-STAT®. The epoc® had 17 instances of out-of-acceptable comparative range results for Hct compared with 0 for i-STAT®. For glucose however, i-STAT® had twice as many out-of-range results for values under 5 mmol/L and three times as many for values greater than or equal to 5 mmol/L. For detailed analyte specific results please see the on-line Additional file 1.

Each individual device was assessed against other devices by the same manufacturer to determine if a small number of devices contributed greater than their fair share of out-of-range results. For the i-STAT® devices, the proportion of out-of-range results by device was 0.0 to 41.7%. One i-STAT® device (CP6) appeared to give more results outside of acceptable comparative ranges than others (Table 3). When i-STAT® CP6 was compared with all other devices, it was found that the odds of getting a value outside of the acceptable comparative range was 3.3 times (95% CI 1.3, 8.3) that of the other devices. For the epoc® devices, the proportion of out-of-range results was 27.3 to 58.8%. There was no one epoc® device that contributed a statistically significant number of out-of-range results when compared with the other epoc® devices (Table 3).

**Table 3:** Summary of out-of-range and in-range results for i-STAT® and epoc® by individual device and by manufacturer

i-STAT®			
Device	Out-of-range^a^	In-range^a^	Proportion Out-of-range by device
CP1	0 (0.0%)	13 (100.0%)	0 (0.0%)
CP2	4 (23.5%)	13 (76.5%)	4 (14.8%)
CP3	4 (23.5%)	13 (76.5%)	4 (14.8%)
CP4	2 (20.0%)	8 (80.0%)	2 (7.4%)
CP5	2 (18.1%)	9 (81.8%)	2 (7.4%)
CP6	15 (41.7%)	21 (58.3%)	15 (55.5%)
Total Out-of-range	27/104 (26.0%)	77/104 (74.0%)	27 (100.0%)
epoc®			
CP1	5 (38.5%)	8 (61.5%)	5 (10.2%)
CP2	6 (31.6%)	13 (68.4%)	6 (12.2%)
CP3	10 (58.8%)	7 (41.2%)	10 (20.4%)
CP4	6 (54.6%)	5 (45.5%)	6 (12.2%)
CP5	3 (27.3%)	8 (72.7%)	3 (6.1%)
CP6	19 (54.3%)	16 (45.7%)	19 (38.8%)
Total Out-of-range	49/106 (46.2%)	57/106 (53.8%)	49 (100.0%)

^a^Out-of-range refers to outside of the a priori laboratory to POCT acceptable Comparative Range and vice versa

### Device usability

All 19 CPs were sent the survey, 17 complete surveys and one partially complete survey were received (94.7% response rate). The respondents had a range of EMS experience from 5 to 32 years (mea*n* = 11.4 years, SD = 6.4) and a range of CP programme experience of 0.2 to 4.1 years (mea*n* = 2.3 years, SD = 1.5)(N.B., the CP programme has been in existence for 4.1 years).

Eleven [11] of 18 (61.1%) respondents chose i-STAT® as their preferred device, with 5 (27.8%) preferring epoc®, and 2 (11%) having no preference. Table 4 outlines the verbatim comments respondents provided on what they liked and did not like about the two devices.

**Table 4:** Summary of community paramedic comments pertaining to device preference

Preference	Comment
i-STAT® (n = 11)	• i-STAT had less errors and easier to use, clean and do QC on. • Easier to use with fewer errors and less time commitment. • Compact and easy. • Ease of use. • Easy to use, uncomplicated, gives the same results as the epoc without the frustration, annoyance & hassle! • Less complicated, easy to clean, no moving parts. • More durable, wait time is after sample introduction, easier to introduce sample and place card in reader. • Easier to use overall, simple is better for continued device confidence, likely won’t require as much re-familiarization. • Felt sturdier. Easier to turn off. Quality control less time consuming. • Slightly easier to learn, handle, clean and use in different environments. • No answer entered.
epoc® (*n* = 5)	• Would not have to be regulated for temperature, no extra cooler to carry around. • More reliable. • I feel like I understand the device better for both trouble shooting and pulling up previous results. • Versatility, no refrigeration of cartridges, and versatility of testing with just one cartridge. • Ease of use, more consistent with no errors, do not have to do daily testing and do not have to refrigerate test cards.
No preference (n = 2)	• No answer entered. • No answer entered.

**Fig. 2:**
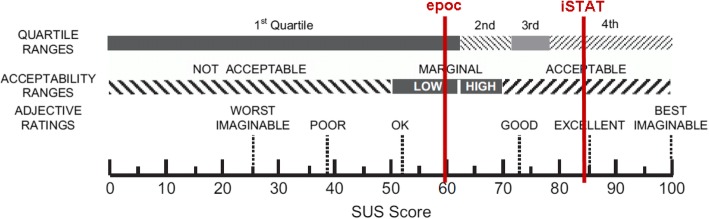
Mean System Usability Scale (SUS) Scores for the epoc® and i-STAT® compared to quartile ranges, acceptability ranges and adjective ratings. Adapted from “An empirical evaluation of the System Usability Scale,” by A. Bangor, P.T. Kortum, and J.T. Miller, 2008, International Journal of Human-Computer Interaction, 24, p. 592. Copyright 2008 by Taylor & Francis Group, LLC

Participants scored the i-STAT® device a mean 24.4 points higher (95% CI 6.9, 42.0.) than the epoc® device. Using the means from the linear mixed effects model (accounting for paramedic experience, CP programme experience and previous experience with a POCT device), the i-STAT® mean score was 84.0 and the epoc® 59.6. Figure 2 compares the mean SUS scores to quartiles for usability developed by Bangor, Kortum and Miller (2008). The epoc® score of 59.6 is in the 1st quartile (lowest) for usability and the i-STAT® score of 84.0 is in the 4th quartile (highest). [12]

**Fig. 3:**
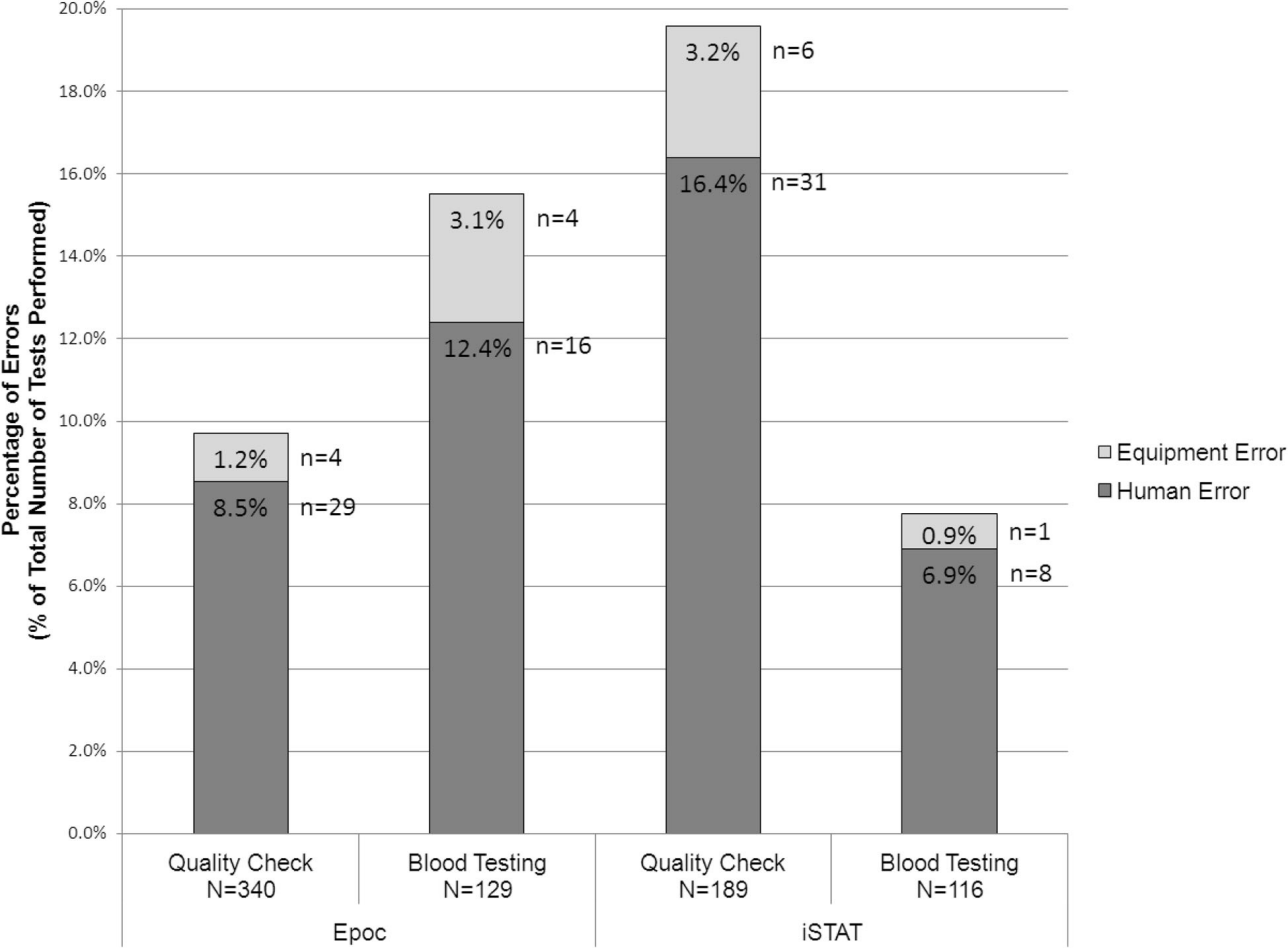
Percentage of logged errors for each POCT device during Quality Check and Patient Blood Testing

Overall, the i-STAT® device logged 46 errors out of 305 tests (15.1%; 95% CI 11.1, 19.1%) compared with the epoc® device, which logged 53 errors out of 469 tests (11.3%; 95% CI 8.4, 14.2%) although these results were not statistically significant (*p* = 0.122). The i-STAT® logged a statistically significant larger proportion of errors during the quality check procedures (37 of 189 tests; 19.6%; 95% CI 13.9, 25.3%) compared with the epoc® (33 of 340 tests; 9.7%; 95% CI 6.6, 12.8%)(*p* = 0.001). However, the i-STAT® experienced fewer errors during the blood testing in the field (9 of 116 tests; 7.8%; 95% CI 2.9, 12.7%) compared with the epoc® (20 of 129 tests; 15.5%; 95% CI 9.3, 21.7%), although these results were not statistically significant (*p* = 0.063) (Fig. 3).

## Discussion

For the device validation, discrepant results for critical range occurred in 0.6% of comparisons, and in 8.4% of comparisons the a priori defined acceptable difference between POCT and the laboratory was exceeded. To rule out possible causes of these results, all out-of-range data for acceptable comparative ranges had a third check for data entry error performed, and no erroneous entries were found. When individual devices by manufacturer were compared, one i-STAT® device accounted for over half of all out-of-range i-STAT® results. This device did not have unusual incidents logged, nor was it exposed to extreme temperatures. All weekly QC testing was completed on the device. The cartridge lots were used by other i-STAT® devices and hence not unique to this device. Four CPs used this device, with two of the CPs using other i-STAT® devices in the study. It is therefore unknown why this particular device would return more out-of-range results compared with the other i-STAT® devices. For epoc®, none of the devices had unusually large numbers of out of–range results compared with each other, although two CPs using one device collected 12 of the 17 out-of-range Hct results on epoc®.

The reasons that the POCT could have returned out-of-range results compared with the laboratory include issues with the device, card or cartridge, or sample preparation. It is difficult to determine retrospectively what may have been the cause. While during weekly QC testing there was the odd failure in one level of QC by one device, there were no trends to suggest that a device was consistently returning out-of-range results. The cards or cartridges were likewise not exposed to any known extreme temperatures.

While the number of results that exceeded the acceptable comparative range was sizeable, few instances of deviations between POCT and laboratory critical values were recorded. These results suggest that the incongruent findings between the laboratory and POCT in most instances were not large enough to affect the identification of a critical situation. Moreover, there were no instances of a missed critical result by the POCT; in all instances the discrepancy was due to the POCT returning the critical value, not the laboratory. The results underscore the importance of proper training, initial device validation, daily and weekly QC checks, split sample testing, and handling and care of POCT devices.

This study also included a comparison of analyte values between the two POCT devices. The rationale for this analysis was for systems that may use devices from both manufacturers. Between the devices (epoc® compared with i-STAT®) there were discrepant critical results in three out of 401 individual comparisons (0.7%), and 38 out of 599 (6.3%) individual comparisons outside of comparative standards. If agencies within the same system use devices from different manufacturers, discrepant results should be anticipated.

This study quantified that CPs will get their results considerably quicker using POCT compared with transportation to laboratory (e.g., an estimated 97 to 163 min), however as POCT may not be capable of running all ordered tests, it should be assumed that POCT will not replace all laboratory testing. For example, in the sample of 108 episodes of care there were 88 episodes (82%) where a white blood cell (WBC) test was also ordered meaning that these episodes would still require transport of blood to the laboratory. Based on these results, it may be reasonable to assume that implementing a POCT programme will not replace transporting blood for laboratory analysis, but rather be an ‘add-on’ process. Other tests were found to have been ordered in the sample of 108 episodes of care that POCT devices are currently unable to test, however the scope of this study did not allow for further analysis of these data. It is not known whether the implementation of a POCT programme may change the ordering habits of physicians. For example, in this sample, physicians were accustomed to ordering through the laboratory analytes they knew were available, and may have ordered WBC because it was convenient or part of a ‘panel’ not because it was required. It could be that with a more limited menu of test options for POCT, physicians modify their ordering practices.

For the device usability, i-STAT® was the preferred device of CPs in this study. The i-STAT® had a lower error rate (than epoc®) during actual patient use but a higher error rate (than epoc®) in QC testing. An issue not automatically logged in the devices’ error logs, but observed in the QC testing, was that the epoc® cartridges needed to be removed and retried 11 out of 27 times (41%) before they would work. Ongoing frustrations with these non-logged issues may be the reason why the users preferred the i-STAT® over the epoc® during the trial. Field observations of POCT use were unable to be conducted during this study, but would provide important information on why the device error rates changed between QC and patient testing and some of the outliers found in the comparison of the POCT with laboratory tests.

The reasons given by people who preferred i-STAT® in general were related to the function of the device. For example, the device was simple, it was easy to clean and use with fewer errors. But in general, the reasons for preferring epoc® were related to the logistics of using the device. For example, the test cards do not need to be refrigerated, there was no daily electronic simulation test and one card performed all the blood tests. There are important differences between these two systems, which should be reviewed prior to selection (see on-line Additional file 1 for a description of the two systems).

### Limitations

This study used a split sample approach where a prehospital POCT result was compared with a laboratory analysis. There are many factors that may have contributed to reported discrepancies between POCT and laboratory results such as timing of blood analysis, methodology, and pre-analytical issues. The timing of the laboratory blood analysis occurred at a different time than the POCT analysis. It took between 17 and 24 min to transport the blood to the laboratory facility and in this time certain analyte levels may have changed. [15] While this can be viewed as a limitation, it also reflects what happens in real-life, where POCT analysis is done before a laboratory analysis. While all attempts were made to train and assess competence in CPs involved in this study, no observational quality assurance was performed to ensure good technique by CPs while out in the field. Differences in technique associated with mixing and storage may have affected individual samples, and individual differences in how a CP tested a sample may explain some of the observed device discrepancy. Finally, data were entered into a Microsoft Excel spreadsheet, which increased the risk of unplanned alterations to the data. To mitigate the limitation of using this program, the spreadsheet was password protected, kept on a limited access shared drive, and only two research associates had access to the file.

## Conclusions

CP programmes can expect clinically valid results from either POCT device for the analytes tested in this study. While discrepant results between the POCT and laboratory were reported, critical range discrepancies occurred in less than 1% of comparisons and there were no instances of a POCT device missing a critical value. Device usability assessments should be considered with any local implementation as the two POCT systems have different strengths.

## Additional file

Additional file 1: **Table S1.** Summary of characteristics of the Abbott i-STAT® and Alere epoc®. **Figure S1.** Results for sodium from i-STAT and epoc compared to gold standard (‘Lab’ – Calgary Lab Services), and between i-STAT and epoc. All results reported in mmol/L. **Figure S2.** Results for potassium from i-STAT and epoc compared to gold standard (‘Lab’ – Calgary Lab Services), and between i-STAT and epoc. All results reported in mmol/L. **Figure S3.** Results for chloride from i-STAT and epoc compared to gold standard (‘Lab’ – Calgary Lab Services), and between i-STAT and epoc. All results reported in mmol/L. **Figure S4.** Results for creatinine from i-STAT and epoc compared to gold standard (‘Lab’ – Calgary Lab Services), and between i-STAT and epoc. All results reported in umol/L. **Figure S5.** Results for hematocrit from i-STAT and epoc compared to gold standard (‘Lab’ – Calgary Lab Services), and between i-STAT and epoc. All results reported in %. **Figure S6.** Results for hemoglobin from i-STAT and epoc compared to gold standard (‘Lab’ – Calgary Lab Services), and between i-STAT and epoc. All results reported in g/L. **Figure S7.** Results for glucose from i-STAT and epoc compared to gold standard (‘Lab’ – Calgary Lab Services), and between i-STAT and epoc. All results reported in mmol/L. **Figure S7.** Results for glucose from i-STAT and epoc compared to gold standard (‘Lab’ – Calgary Lab Services), and between i-STAT and epoc. All results reported in mmol/L. (DOCX 773 kb)

## Abbreviations

Cl: Chloride
CPs: Community paramedics
Crea: Creatinine
EMS: Emergency medical services
ePCR: Electronic patient care record
Glu: Glucose
Hct: Hematocrit
Hgb: Hemoglobin
K: Potassium
Na: Sodium
POCT: Point of care testing
QC: Quality control
SUS: System usability scale
WBC: White blood cell
