# Supplementary material for: Community paramedic point of care testing: validity and usability of two commercially available devices
Source: BMC Emerg Med. 2019 May 2;19:30. doi: 10.1186/s12873-019-0243-4 (PMC6498549; doi:10.1186/s12873-019-0243-4)
Supplement: Supplementary file 1 — Table S1. Summary of characteristics of the Abbott i-STAT® and Alere epoc®. Figure S1. Results for sodium from i-STAT and epoc compared to gold standard (‘Lab’ – Calgary Lab Services), and between i-STAT and epoc. All results reported in mmol/L. Figure S2. Results for potassium from i-STAT and epoc compared to gold standard (‘Lab’ – Calgary Lab Services), and between i-STAT and epoc. All results reported in mmol/L. Figure S3. Results for chloride from i-STAT and epoc compared to gold standard (‘Lab’ – Calgary Lab Services), and between i-STAT and epoc. All results reported in mmol/L. Figure S4. Results for creatinine from i-STAT and epoc compared to gold standard (‘Lab’ – Calgary Lab Services), and between i-STAT and epoc. All results reported in umol/L. Figure S5. Results for hematocrit from i-STAT and epoc compared to gold standard (‘Lab’ – Calgary Lab Services), and between i-STAT and epoc. All results reported in %. Figure S6. Results for hemoglobin from i-STAT and epoc compared to gold standard (‘Lab’ – Calgary Lab Services), and between i-STAT and epoc. All results reported in g/L. Figure S7. Results for glucose from i-STAT and epoc compared to gold standard (‘Lab’ – Calgary Lab Services), and between i-STAT and epoc. All results reported in mmol/L. Figure S7. Results for glucose from i-STAT and epoc compared to gold standard (‘Lab’ – Calgary Lab Services), and between i-STAT and epoc. All results reported in mmol/L. (DOCX 773 kb) [file 12873_2019_243_MOESM1_ESM.docx]

| **Supplementary Table 1:** Summary of characteristics of the Abbott i-STAT® and Alere epoc®. | | | | | | |
| --- | --- | --- | --- | --- | --- | --- |
|  | **Device operating temperature requirements** | **Device transport requirements** | **Device power requirements** | **Test cartridge transport requirements**  **(in CP vehicle)** | **Test cartridge temperature storage requirements** | **Test card / cartridges** |
| **epoc** | Yes  (15-30°C)  Device will lock out if out of temperature range | Requires protection from excessive movement / dropping  If the device is misused or dropped the epoc will give an error. | Battery (rechargeable)  Should recharge battery daily using AC plug in power source, to ensure device is ready for use.  On a full charge the epoc is able to run 50 patient samples, the epoc system will give the user a message by the LED lights blinking to know when it is time to charge as well if it is almost depleted, the epoc will have a red banner indicating ‘charge battery’ | Must be protected against excessive shock (dropping, throwing, shaking)  Must be protected against temperatures outside of  15-30°C during storage and transport  Consider temperature monitoring device for transport container | Yes, (room temperature 15-30°C) at all times. | One test card for all analytes |
| **i-STAT** | Yes  (16-30°C)  Device will lock out if out of temperature range | Excessive movement / dropping well tolerated during transport. | Battery powered by two 9V lithium batteries for a minimum of 250 patient samples depending on type of test run  The analyzer can also be powered by a nickel-metal-hydride rechargeable battery. Requires a charge once every three months. | Must be protected against temperatures outside 18-30°C during storage and transport  Consider temperature once removed from cold storage (2-8 °C) | Yes, (cold chain 2-8°C)  Or if room temperature 18-30°C, cartridge must be used within 14 days.  Requires refrigeration to maintain cold chain with temperature monitoring capabilities | Large test menu, but may require multiple cartridges based on user needs |
| **Laboratory** | n/a | n/a | n/a | n/a | n/a | n/a |

| **Supplementary Table 1 (continued):** Summary of characteristics of the Abbott i-STAT® and Alere epoc®. | | | | | |
| --- | --- | --- | --- | --- | --- |
|  | **Test menu** | **QC fluid storage requirements** | **QC testing frequency*** | **Device validation**  **As per CLS & AHS standards** | **Transport time to specimen drop off** |
| **epoc** | pH, pCO2, pO2  Sodium, Potassium, Ionized Calcium, Chloride  Glucose  Lactate  Creatinine  Hematocrit  Calculated parameters:  Hemoglobin, cHCO3-, TCO2, BE(ecf), BE(b), cSO2, eGFR, AGap, AGapK | Cold chain (2-8°C)  Requires refrigeration with temperature monitoring capabilities | - Weekly per device - Daily per device - Monthly split sample for creatinine - Weekly split sample for blood gases - Requires two test cartridges and two ampoules of QC fluid per week per device - Each new test cartridge lot# must be validated using two levels of QC fluid | Must validate all devices for all analytes prior to use, using lab-based split sample, QC fluid, and cal-ver fluid | n/a |
| **i-STAT** | Sodium, Potassium, Chloride, TCO2, Anion Gap, Ionized Calcium, Glucose, Urea Nitrogen, Creatinine, Lactate  Hematocrit, Hemoglobin a  pH, PCO2, PO2, TCO2a, HCO3a, Base Excess (BE)a, sO2a  ACT Kaolin, ACT Celite®, PT/INR, aPTT end of 2017,  ß-hCG, Cardiac Markers, CK-CK-MB  BNP, cTnl, High Sene. cTnl 2018 | Cold chain (2-8°C)  Requires refrigeration with temperature monitoring capabilities | - Weekly per device - Daily per device - Monthly split sample for creatinine - Weekly split sample for blood gases - Requires two test cartridges and two ampoules of QC fluid per week per device - Each new test cartridge lot# must be validated using two levels of QC fluid | Must validate all devices for all analytes prior to use, using lab-based split sample, QC fluid, and cal-ver fluid | n/a |
| **Laboratory** | All analytes are available. | n/a | All QC completed by CLS | n/a | The mean time to transport a specimen to CLS was 19.7 minutes (SD 14.1; 95% CI 17.0, 22.4) |
| Note: CLS= Calgary Lab Services; QC=Quality control.  * As per standards set by Calgary Lab Services | | | | | |

| **Supplementary Table 1 (continued):** Summary of characteristics of the Abbott i-STAT® and Alere epoc®. | | | | |
| --- | --- | --- | --- | --- |
|  | **Time to results** | **Start-up equipment** | **Training** | **Competency maintenance**  **as per CLS standards** |
| **epoc** | Results available on-scene  30 seconds after introduction of sample into device | Device(s)  Test cards and solutions for validation (QC, calibration-verification, patient split samples)  Storage and transport packaging (fridge, thermal containers)  Does not include specimen collection equipment | Two hours in classroom setting for device, plus evaluation | At least three yearly |
| **i-STAT** | Results available on-scene  120 second after introduction of test cartridge into device | Device(s), Test cards and solutions for validation (QC, calibration-verification, patient split samples)  Storage and transport packaging (fridge, thermal containers)  Electronic simulator  Does not include specimen collection equipment | Two hours in classroom setting, plus evaluation | At least three yearly |
| **Laboratory** | The mean time between the mean of the two POCT device results being available, to the result being available from CLS was 129.7 minutes (SD 169.7; 95% CI 96.9, 162.6). | Specimen transport box  In accordance with Canadian Transportation of Dangerous Goods Act and Regulations (TDG)  Does not include specimen collection equipment | One hour in classroom to review Laboratory specimen collection / drop off procedure | n/a |
| Note: QC=Quality control.  * As per standards set by Calgary Lab Services | | | | |

| **Supplementary Table 1 (continued):** Summary of characteristics of the Abbott i-STAT® and Alere epoc®. | | | |
| --- | --- | --- | --- |
|  | **Results management** | **Blood sample quantity required for test** | **Validity of results** |
| **epoc** | Results not currently able to be uploaded onto provincial patient electronic healthcare record (Netcare)  Results manually entered into EMS patient care record  Results only viewable by CP or by physician if patient care record faxed to referring physician  Additional software can be purchased to have results downloaded into a central server | Small volume of blood required <1 ml | Valid results for all analytes assessed in this study |
| **i-STAT** | Results are able to be transmitted to Netcare, however requires data management and middleware and interface to LIS (Meditech, Sunnytech, etc.). This is an expense to the user.  Results manually entered into EMS patient care record  Results only viewable by CP or by physician if patient care record faxed to referring physician  Additional software can be purchased to have results downloaded into a central server | Small volume of blood required <1 ml | Valid results for all analytes assessed in this study |
| **Laboratory** | Once verified, results posted to the CLS database (Cerner Millennium) and then posted to Netcare.  Results viewable by any healthcare provider involved in patient’s care via Netcare. | Varies, but generally minimum volumes required <2 ml | Gold standard |
|  | | | |

S1 TOP

**Supplementary Figure 1:** Results for sodium from i-STAT and epoc compared to gold standard (‘Lab’ – Calgary Lab Services), and between i-STAT and epoc. All results reported in mmol/L.

Upper graphs illustrate the critical range defined as less than 120 and greater than 155 mmol/L (red lines), with the black line denoting perfect agreement. Areas of disagreement between device and reference method are shaded in red.

Lower graphs illustrate the acceptable comparative range defined as less than -4 and greater than 4 mmol/L (red lines), with the black line denoting mean of the difference.

S2 TOP

**Supplementary Figure 2:** Results for potassium from i-STAT and epoc compared to gold standard (‘Lab’ – Calgary Lab Services), and between i-STAT and epoc. All results reported in mmol/L.

Upper graphs illustrate the critical range defined as less than 2.5 and greater than 6 mmol/L (red lines), with the black line denoting perfect agreement. Areas of disagreement between device and reference method are shaded in red.

Lower graphs illustrate acceptable comparative range defined as less than -0.3 and greater than 0.3 mmol/L (red lines), with the black line denoting mean of the difference.

S3 TOP

**Supplementary Figure 3:** Results for chloride from i-STAT and epoc compared to gold standard (‘Lab’ – Calgary Lab Services), and between i-STAT and epoc. All results reported in mmol/L.

Upper graphs – there is no defined critical range; the black line denotes perfect agreement.

Lower graphs illustrate acceptable comparative range defined as less than -5% and greater than 5% of reference method (red lines), with the black line denoting mean of the difference.

S4 TOP

**Supplementary Figure 4:** Results for creatinine from i-STAT and epoc compared to gold standard (‘Lab’ – Calgary Lab Services), and between i-STAT and epoc. All results reported in umol/L

Upper graphs – there is no defined critical range; the black line denotes perfect agreement.

Lower graphs illustrate the acceptable comparative range defined as less than -30 and greater than 30 umol/L of reference method (red lines), with the black line denoting mean of the difference.

S5 TOP

**Supplementary Figure 5:** Results for hematocrit from i-STAT and epoc compared to gold standard (‘Lab’ – Calgary Lab Services), and between i-STAT and epoc. All results reported in %.

Upper graphs – there is no defined critical range; the black line denotes perfect agreement.

Lower graphs illustrate the acceptable comparative range defined as less than -6.0% and greater than 6.0% of reference method (red lines), with the black line denoting mean of the difference.

S6 TOP

**Supplementary Figure 6:** Results for hemoglobin from i-STAT and epoc compared to gold standard (‘Lab’ – Calgary Lab Services), and between i-STAT and epoc. All results reported in g/L.

Upper graphs illustrate the critical range defined as less than 70 g/L (red lines), with the black line denoting perfect agreement. Areas of disagreement between device and reference method are shaded in red.

Lower graphs – there is no acceptable comparative range for this analyte as it is a calculation based on hematocrit; the black line denotes mean of the difference.

S7 TOP

**Supplementary Figure 7:** Results for glucose from i-STAT and epoc compared to gold standard (‘Lab’ – Calgary Lab Services), and between i-STAT and epoc. All results reported in mmol/L.

Upper graphs illustrate the critical range defined as less than 2.6 and greater than 24.9 mmol/L (red lines), with the black line denoting perfect agreement. Areas of disagreement between device and reference method are shaded in red.

Middle graphs illustrate acceptable comparative range for observations under 5 mmol/L. The acceptable comparative range is defined as less than -0.3 and greater than 0.3 mmol/L (red lines), with the black line denoting mean of the difference.

Lower graphs illustrate the acceptable comparative range for observations greater than 5 mmol/L. The acceptable comparative range is defined as less than -10% and greater than 10% (red lines), with the black line denoting mean of the difference.
